# Supplementary material for: Are Users Good Assessors of Social Dominance in Domestic Horses?
Source: Animals (Basel). 2024 Jul 7;14(13):1999. doi: 10.3390/ani14131999 (PMC11240818; doi:10.3390/ani14131999)
Supplement: Supplementary file 1 [file animals-14-01999-s001.zip › animals-3057895-supplementary.pdf]

Table S1. Survey of the raters

**Survey item**	**Rater**							
	A	B	C	D	E	F	G	H
1. How long have you been an instructor?	25				22	20		30
2. During each stay at the stable, how much time (in minutes, on average) do you spend observing the horses (not including time of teaching riding)?	20	25	25	20	15	10	25	30
3. Does it happen that horses are aggressive towards each other while preparing for riding?	1	1	1	1	1	1	1	1
4. Do horses sometimes become aggressive towards each other while riding?	1	1	1	1	1	1	1	1
5. Does it happen that horses are aggressive towards each other while on the paddock?	1	1	1	1	1	1	1	1
6. Is it easy to identify aggressive horses?	1	1	1	1	1	1	1	1
7. Is it easy to identify submissive horses?	1	1	1	1	1	1	1	1
8. When selecting horses for riding, do you take into account primarily the behaviour of the horses towards each other or rather the level of the rider?	3		3		3	2		3
9. Do you determine the order of horses riding in a single file?	1		1		1	1		1
10. Do you have a scientific basis for knowledge of horse ethology?	1	1	1	1	1	1		1
11. Did you gain knowledge about horse behaviour from equine 'behaviourists'?	1	1	1	1	1	1		0

1: yes; 2: the level of the rider; 3: both the level of the rider and the behaviour of the horse; blank: the rater is not an instructor
